# Acquired factor VII inhibitor associated with primary central nervous system Lymphoma: A case report

**DOI:** 10.1002/jha2.482

**Published:** 2022-06-07

**Authors:** Vanshika Goyal, Giselle Salmasi, Andrew D. Leavitt, James L. Rubenstein, Rahul Banerjee

**Affiliations:** ^1^ Department of Neurobiology Physiology, and Behavior, University of California Davis California USA; ^2^ Division of Hematology/Oncology Department of Medicine University of California San Francisco California USA; ^3^ Department of Laboratory Medicine University of California San Francisco San Francisco California USA; ^4^ Division of Medical Oncology Department of Medicine University of Washington Seattle Washington USA

**Keywords:** case report, CNS lymphoma, coagulation, factor VII, lymphoma

## Abstract

Paraneoplastic coagulopathies are uncommon in patients with lymphoma. We report the first case of an acquired coagulopathy in a patient with isolated primary central nervous system lymphoma (PCNSL) demonstrating large‐cell histology. In our patient, a paraneoplastic factor VII inhibitor significantly delayed a diagnostic lumbar puncture despite fresh frozen plasma and inactivated prothrombin complex concentrate. While her coagulopathy was effectively overcome with recombinant activated factor VIIa and subsequently with lymphoma‐directed therapy, her delayed diagnosis likely contributed to a poor outcome. Our case highlights the importance of rapidly identifying and correcting paraneoplastic coagulopathies when PCNSL is suspected.

1

Paraneoplastic manifestations of lymphoma include hematologic abnormalities and neurological deficits [1]. As summarized in Table 1, paraneoplastic coagulopathies have rarely been reported in the setting of systemic involvement by lymphoma [2, 3, 4, 5, 6, 7, 8]. We report the first case of a paraneoplastic coagulopathy in isolated primary central nervous system lymphoma (PCNSL). In our patient's case, an acquired factor VII inhibitor led to a prolonged prothrombin (PT) time and international normalized ratio (INR) that hindered the safety of a diagnostic lumbar puncture (LP). Our case highlights the importance of suspecting and correcting such coagulopathies rapidly to avoid diagnostic delays and to establish optimal treatment.

**TABLE 1:** Published cases of paraneoplastic coagulopathy in patients with lymphoma

**Reference**	**Type of lymphoma**	**Inhibited factor**	**Bleeding at presentation**	**Clinical Outcome**
Gesierich 2000 [2]	CLL	VIII	Cutaneous	*N/A*
Rungjirajittranon 2021 [8]	LPL	X	Menorrhagia	Remission
Aljohani 2014 [4]	MCL	V	Hematuria	*N/A*
Nixon 2016 [6]	MCL	XIII	Menorrhagia	Remission
Meenhuis 2015 [5]	MZL	X	Cutaneous	Remission
Naismith 2021 [7]	MZL	VII	*None*	Remission
Lee 2001 [3]	*N/A*	II	Cutaneous	Remission
Goyal 2022*	PCNSL	VII	*None*	Death

* Refers to the case being presented in this report.

Abbreviations: CLL, chronic lymphocytic leukemia; LPL, lymphoplasmacytic lymphoma; MCL, mantle cell lymphoma; MZL, marginal zone lymphoma; N/A, not available; PCNSL, primary central nervous system lymphoma.

Our case involves a 64‐year‐old female who initially presented to a different hospital with a 1‐month history of worsening right leg weakness. Her medical history was notable for hypertension and diabetes but negative for excessive bleeding or bruising. At initial presentation, she required a cane to ambulate but was fully alert and oriented. Brain magnetic resonance imaging (MRI) showed several contrast‐enhancing lesions in her left frontal lobe and basal ganglia. Relevant bloodwork included an elevated PT of 21.3 s (reference interval 11.8–14.8 s) with INR of 2.1 (reference interval [RI] 0.9–1.2). The partial thromboplastin time (PTT) was normal at 23.5 s (RI 22.6–34.5 s).

Given the patient's primary residence in Mexico, she was empirically treated with albendazole for presumed neurocysticercosis. However, she became progressively obtunded over the following three weeks. Serologic testing for *Taenia solium*, *Cryptococcus neoformans*, *Toxoplasma gondii*, and the human immunodeficiency virus (HIV) were negative. The patient's hospital team subsequently recommended LP to rule out a malignancy. However, the patient's INR did not improve despite several doses of vitamin K, fresh frozen plasma (FFP), and inactivated four‐factor prothrombin complex concentrate (PCC). As such, several attempts to perform an LP or brain biopsy were deferred.

Her family ultimately requested her transfer to our hospital approximately three months after symptom onset. On arrival to our hospital, the patient remained obtunded. Repeat MRI brain showed increased disease burden compared to initial imaging, both by size and T2‐weighted Fluid‐Attenuated Inversion Recovery (FLAIR) conspicuity. Positron emission tomography/computed tomography (PET‐CT) imaging and bone marrow biopsy were both unremarkable. PT was 19.7 s (INR 1.7), and mixing studies were suggestive of an inhibitor. Subsequent testing revealed an IgM lambda paraprotein of 0.8 g/dl and decreased factor VII activity of 24% (RI 54–169%). Factor II, V, and X activity levels were 59% (RI 81–127%), 99% (RI 67–154%), and 66% (RI 71–143%), respectively. Given the urgency of making a diagnosis, the patient received 15 μg/kg of recombinant activated factor VII (rFVIIa) the following day. Thirty minutes after rFVIIa administration, the patient's PT/INR normalized (11.9 s and 0.9, respectively) and bedside LP was performed uneventfully.

Cerebrospinal fluid flow cytometry revealed abnormal B‐cells with high forward scatter and CD10 expression without CD5 or CD23 expression, most compatible with aggressive large‐cell histology. The patient was immediately started on the methotrexate/temozolomide/rituximab (MTR) regimen for confirmed PCNSL, with temozolomide omitted given her obtundation and inability to take pills [9]. Repeat brain MRI 4 weeks after treatment initiation showed an interval response. PT/INR normalized as shown in Figure 1, repeat factor VII activity was 72%, and the patient's paraprotein decreased to 0.2 g/dl. Unfortunately, the patient's mental status did not improve with several cycles of MTR. Her family opted to transition to hospice care, and the patient passed away six months after symptom onset. Her family provided consent for her case to be published.

**FIGURE 1:**
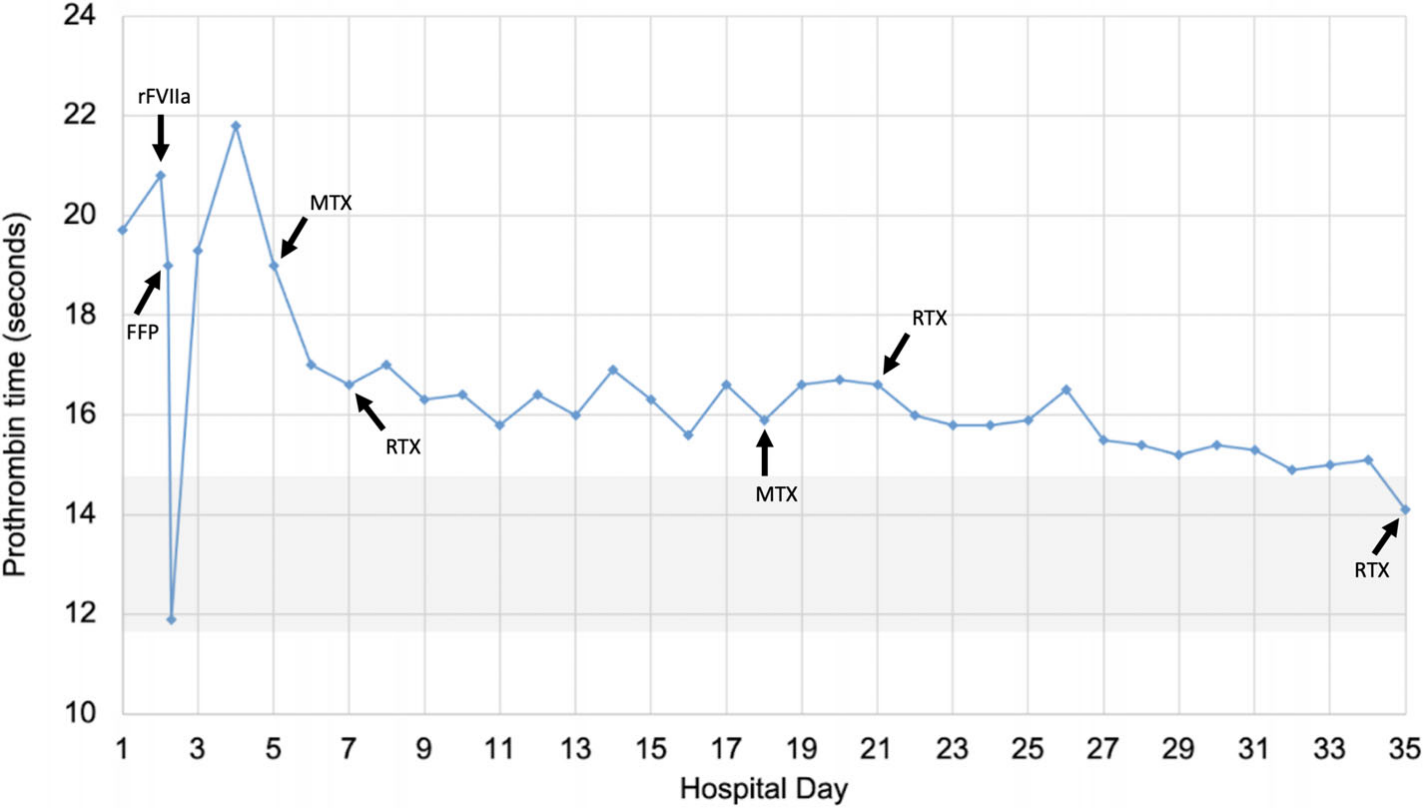
Time course of patient's coagulopathy and treatment. Hospital days represent days since arrival to our facility. The reference interval for prothrombin time at our institution is shaded in grey. FFP, fresh frozen plasma; MTX, methotrexate; rFVIIa, recombinant activated factor VII; RTX, rituximab

This case is unique in two respects. First, to our knowledge, this is the first report of an acquired coagulopathy in the setting of PCNSL without systemic involvement. While it is possible that the patient's paraprotein was unrelated to her lymphoma, bone marrow biopsy showed no evidence of a concomitant plasma cell dyscrasia. Similarly, although IgM paraproteins are classically found in lymphoplasmacytic lymphoma (LPL), their presence in PCNSL has previously been reported [10, 11]. Additionally, the high forward scatter and CD10 positivity seen in our patient's case would be unusual for LPL. A second unique component of our case is the acquired inhibition of factor VII, a rare phenomenon with fewer than 50 reported cases [12]. A paraneoplastic factor VII inhibitor in lymphoma has only been reported once previously, in a patient with splenic marginal zone lymphoma and marrow involvement [7]. Of note, paraproteins may mildly prolong PT in a non‐specific manner [13]. However, the isolated factor VII deficiency in our patient's case—coupled with an improvement in factor VII activity and paraproteinemia with lymphoma‐directed therapy—suggest a specific link. In the absence of binding assays or epitope mapping, we are unable to definitively prove this association.

Our case highlights the unique diagnostic challenge that paraneoplastic coagulopathies create with regard to PCNSL. In our patient's case, standard coagulopathy workup revealed the presence of an antibody‐based inhibitor of factor VII [14]. However, over three months elapsed between symptom onset and treatment initiation. Previous LP attempts had been repeatedly delayed by elevated INR levels unresponsive to FFP and inactivated PCC, highlighting the importance of activated rFVII in this setting. We chose a low dose of rFVIIa (15 μg/kg) to minimize the risk of thrombotic events in this immobile patient with an untreated malignancy, which successfully reversed her coagulopathy.

In conclusion, paraneoplastic coagulopathies are rare but possible in PCNSL. Importantly, our patient was fully alert and oriented at her initial presentation to a healthcare setting. Whether establishing a diagnosis and initiating lymphoma‐directed therapy at presentation would have prevented her neurological decline and death is unknown. However, this possibility reinforces the importance of prompt and aggressive management to correct coagulopathy in patients with lymphoma, particularly when the diagnosis is not yet established.

## ETHICS STATEMENT

Informed consent was obtained for the patients case to be reported.

## FUNDING INFORMATION

The authors received no specific funding for this work.

## CONFLICT OF INTEREST

VG: No interests to disclose. GS: No interests to disclose. ADL: BioMarin, advising; Bioproducts Laboratory; Catalyst Biosciences, advising; Dova Pharmaceuticals, advising; Johnson & Johnson, shareholder; Merck, consulting. JLR: BMS, research support; Genentech, research support. RB: Curio Science, honoraria; Eradigm Consulting, consulting; i3 Health, advising; Guidepoint Global, consulting; Sanofi Pasteur, consulting; SparkCures, consulting.

## Data Availability

The data that support the findings of this case report are available from the senior author, Dr. Banerjee, upon reasonable request.
